# Three new species of the millipede genus *Tylopus* Jeekel, 1968 from Thailand, with additional notes on the species described by Attems (Diplopoda, Polydesmida, Paradoxosomatidae)

**DOI:** 10.3897/zookeys.435.8286

**Published:** 2014-08-18

**Authors:** Natdanai Likhitrakarn, Sergei I. Golovatch, Somsak Panha

**Affiliations:** 1 Division of Plant Protection, Faculty of Agricultural Production, Maejo University, Chiang Mai, 50290, Thailand; 2 Institute for Problems of Ecology and Evolution, Russian Academy of Sciences, Leninsky pr. 33, Moscow 119071, Russia; 3 Animal Systematics Research Unit, Department of Biology, Faculty of Science, Chulalongkorn University, Bangkok, 10330, Thailand

**Keywords:** Millipede, *Tylopus*, taxonomy, new species, key, Thailand

## Abstract

*Tylopus* currently comprises 55 species, including three new from Thailand: *T. corrugatus* **sp. n.**, *T. trigonum* **sp. n.** and *T. parahilaroides* **sp. n.** A new distribution map and an updated key to all 29 species of *Tylopus* presently known to occur in Thailand are given. Illustrated redescriptions of all four Indochinese *Tylopus* species described by Carl Attems are also provided, based on type material.

## Introduction

The Southeast Asian millipede genus *Tylopus* Jeekel, 1968 is one of the most speciose not only in the mainly Asian tribe Sulciferini, but also in the whole family Paradoxosomatidae. The latter is probably the largest in the entire class Diplopoda, dominating the millipede fauna of Indo-Australia (Jeekel 1968) and currently comprising nearly 200 genera and over 1,000 species (Nguyen and Sierwald 2013; authors’ records). At the moment, all 52 constituent species of *Tylopus* range from southern China, through Laos, to Myanmar, western Thailand and southern Vietnam. Since the thorough reviews of the genus by Golovatch and Enghoff (1993) and Likhitrakarn et al. (2010), both of which focused on the fauna of Thailand, Nguyen (2012) provided a synopsis of and a key to all 18 species of *Tylopus* occurring in Vietnam, while Golovatch (2013, 2014) summarized all six congeners recorded in southern China.

The present paper is an updated review of all 29 *Tylopus* currently known from Thailand (Table 1), including three new congeners. In addition, all four Indochinese *Tylopus* species described by Carl Attems are redescribed and illustrated, based on the types kept in the collection of the Naturhistorisches Museum Wien, Austria.

**Table 1.** *Tylopus* species recorded in Thailand (Pocock 1895, Hoffman 1973, Golovatch and Enghoff 1993, Likhitrakarn et al. 2010).

No.	Species	Locality
1	*Tylopus affinis* Golovatch & Enghoff, 1993	Doi Pui, summit (1,650 m), Doi Suthep National Park; Mae Chaem road (1,700 m); main road (1,900 m), Doi Inthanon National Park, Chiang Mai Province.
2	*Tylopus allorugosus* Golovatch & Enghoff, 1993	Siripum Waterfall (1,300–1,400 m); Mae Chaem road (1,694–1,700 m); main road (2,200–2,500 m); Doi Inthanon National Park; Doi Pui, summit (1,650 m), Doi Suthep National Park, Chiang Mai Province.
3	*Tylopus amicus* Golovatch & Enghoff, 1993	northwestern Fang District (1,550–1,750 m), Doi Pha Hom Pok National Park, Chiang Mai Province.
4	*Tylopus asper* Golovatch & Enghoff, 1993	Doi Inthanon National Park (1,500 m), Chiang Mai Province.
5	*Tylopus baenzigeri* Golovatch & Enghoff, 1993	near stream (1,100 m); Doi Pui-Chang Khian (1,400–1,500 m), Doi Suthep National Park, Chiang Mai Province.
6	*Tylopus bispinosus* Likhitrakarn, Golovatch, Prateepasen & Panha, 2010	near Umphang City (492 m); Doi Hua Mod (900 m), Umphang District, Tak Province.
7	*Tylopus coriaceus* Golovatch & Enghoff, 1993	Phu Kheio (1,000 m), Chaiyapum Province.
8	*Tylopus corrugatus* sp. n.	Doi Inthanon National Park (1,700 m), Chiang Mai Province.
9	*Tylopus degerboelae* Golovatch & Enghoff, 1993	forest near stream (1,000 m); Doi Pui road (1,000–1,100 m); Siriphum Waterfall (1,298 m); evergreen forest (1,300–1,500 m), Doi Suthep National Park; main road (1,500–1,600), Doi Inthanon National Park; limestone area, Doi Chiang Dao; Doi Phatang, Wiang Kaen District, Chiang Mai Province.
10	*Tylopus doriae* (Pocock, 1895)	Doi Suthep National Park (1,400–1,500 m), Chiang Mai Province.
11	*Tylopus extremus* Likhitrakarn, Golovatch, Prateepasen & Panha, 2010	Doi Phahom Pok National Park, Fang District, Chiang Mai Province.
12	*Tylopus grandis* Likhitrakarn, Golovatch, Prateepasen & Panha, 2010	near Pha Mon Cave; Mae Lana crossroad, Pangmapha District, Mae Hong Son Province.
13	*Tylopus haplorugosus* Golovatch & Enghoff, 1993	main road (1,694–1,900 m), Doi Inthanon National Park, Chiang Mai Province.
14	*Tylopus hoffmani* Golovatch & Enghoff, 1993	summit (1,600 m), Doi Suthep National Park, Chiang Mai Province.
15	*Tylopus jeekeli* Golovatch & Enghoff, 1993	Siripum Waterfall (1,200–1,300 m), Doi Inthanon National Park; Doi Suthep National Park (1,298 m), Chiang Mai Province.
16	*Tylopus pallidus* Golovatch & Enghoff, 1993	northwest of Fang (1,550–1,750 m); Doi Pha Hom Pok, Chiang Mai Province.
17	*Tylopus parajeekeli* Likhitrakarn, Golovatch, Prateepasen & Panha, 2010	summit (2,520 m), Doi Inthanon National Park, Chiang Mai Province.
18	*Tylopus perarmatus* Hoffman, 1973	east slope (1,000–1,275 m); Mahidol Waterfall (1,250–1,500 m), Doi Suthep National Park; Siripum Waterfall (1,300–1,400 m); Vajirathan Waterfall (750 m), Doi Inthanon National Park; Doi Phatang, Wiang Kaen District; limestone cave (500 m), Doi Chiang Dao, Chiang Mai Province. sandy bank of stream (900 m), ca 8 km east of Ban Huai Kaeo, Thoen District; Thum Pha Thai, Ngao District, Lampang Province. Ban Pang Rim Kon, Mueang Chiang Rai District; Phucheefah, Thoeng District; Doi Pha Tang, Wiang Kaen District, Chiang Rai Province. Nam Min Waterfall, Chiang Kham District, Phayao Province. Tham Pha Nang Khoi (275 m), Rong Kwang District, Phrae Province. Ton Tong waterfall, Pua District, Nan Province.
19	*Tylopus perplexus* Golovatch & Enghoff, 1993	northwest of Fang (1,550–1,750 m); Doi Pha Hom Pok, Chiang Mai Province.
20	*Tylopus poolpermorum* Golovatch & Enghoff, 1993	northwest of Fang (1,550–1,750 m); Doi Pha Hom Pok, Chiang Mai Province.
21	*Tylopus prosperus* Golovatch & Enghoff, 1993	main road (2,200 m); summit (2,500 m), Doi Inthanon National Park, Chiang Mai Province.
22	*Tylopus pulvinipes* Golovatch & Enghoff, 1993	Tong Kamang Noi, forest (1,000 m); Phu Kheio, Chaiyaphum Province.
23	*Tylopus rugosus* Golovatch & Enghoff, 1993	Chiang Dao (1,800 m); Buathong Waterfall Forest Park (510 m), Phrao District, Chiang Mai Province.
24	*Tylopus semirugosus* Golovatch & Enghoff, 1993	Ban Mussoe, Mae Sot District, Tak Province.
25	*Tylopus similirugosus* Golovatch & Enghoff, 1993	Doi Suthep National Park (1,000 m); same locality (1,400–1,500 m), Chiang Mai Province.
26	*Tylopus parahilaroides* sp. n.	Phuluang Wildlife Sanctuary (1,486 m), Phuluang District, Loei Province.
27	*Tylopus subcoriaceus* Golovatch & Enghoff, 1993	near stream (1,000 m); evergreen forest (1,100 m), Doi Suthep National Park, Chiang Mai Province.
28	*Tylopus trigonum* sp. n.	Pa Wai Waterfall (804 m), Umphang District, Tak Province.
29	*Tylopus veliger* Likhitrakarn, Golovatch, Prateepasen & Panha, 2010	Ton Tong Waterfall (1,128 m), Pua District, Nan Province.

## Material and methods

New material was collected in northern Thailand and southern Laos from 2011 to 2013 by SP and members of the Animal Systematics Research Unit, Chulalongkorn University. Live animals were photographed in the laboratory shortly before fixing. Specimens were preserved in 75% ethanol, and morphological investigations were carried out in the laboratory using an Olympus stereomicroscope. Scanning electron micrographs (SEM) of gonopods coated with gold were taken using a JEOL, JSM–5410 LV microscope, and the gonopods removed from stubs and returned to alcohol after examination. Digital images of preserved specimens were taken in the laboratory and assembled using the “Cell^D^” automontage software of the Olympus Soft Imaging Solution GmbH package. In addition, line drawings of gonopod characters were also prepared. Type material of the Attemsian congeners housed in the Vienna Museum was photographed with a Dino-Eye Eyepiece USB Camera AM423X, the digital images assembled using the automontage software technique, and the gonopods redrawn. Holotypes of the three new species, as well as most of the paratypes are housed in the Museum of Zoology, Chulalongkorn University (CUMZ), Bangkok, Thailand, a single duplicate paratype being donated to the collection of the Naturhistorisches Museum Wien, Austria (NHMW), as indicated in the text.

Collecting site positions and elevations were determined by GPS using the WGS84 datum.

In the catalogue sections, D stands for the original description, subsequent descriptive notes or appearance in a key, R for a subsequent record or records, and M for a mere mention.

## Taxonomy

### Family Paradoxosomatidae Daday, 1889
Subfamily Paradoxosomatidae Daday, 1889
Tribe Sulciferini Attems, 1898
Genus *Tylopus* Jeekel, 1968

#### Tylopus corrugatus sp. n.

Taxon classificationAnimaliaPolydesmidaParadoxosomatidae

http://zoobank.org/BF1AF28F-1392-44B1-A117-7C8F47BDF77A

Figs 1 – 3

##### Holotype.

♂ (CUMZ), Thailand, Chiang Mai Province, Chom Thong District, Doi Inthanon National Park, 1,700 m a.s.l., 18°31'55"N, 98°29'30"E, 20.12.2013, leg. N. Likhitrakarn & S. Chaiwong.

##### Paratypes.

2 ♂, 6 ♀, 2 juveniles (CUMZ), 1 ♂ (NHMW), same locality, together with holotype. 1 ♂, 1 ♀ (CUMZ), same locality, 25.01.2013, leg. N. Likhitrakarn.

##### Name.

To emphasize the clearly wrinkled postcollum metaterga.

##### Diagnosis.

Differs from congeners mainly in the very strongly developed paraterga with evident oblong ridges. Gonopod process **h** prominent, hook-shape, much longer than solenophore.

##### Description.

Length 15.5–18.2 (♂) or 16.5–21.0 mm (♀), width of midbody pro- and metazonae 1.50–1.70 and 1.95–2.15 mm (♂) or 1.55–2.0 and 2.0–2.5 mm (♀), respectively.

**Figure 1.**
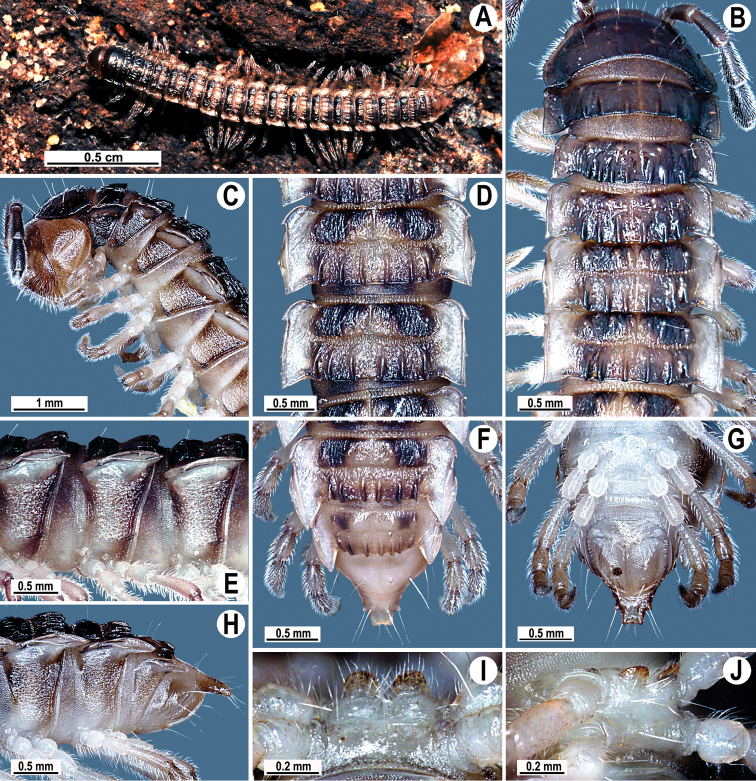
*Tylopus corrugatus* sp. n., ♂ holotype; **A** habitus, live coloration **B, C** anterior part of body, dorsal and lateral views, respectively **D, E** segments 10 and 11, dorsal and lateral views, respectively **F–H** posterior part of body, dorsal, ventral and lateral views, respectively **I, J** sternal cones between coxae 4, subcaudal and sublateral views, respectively.

Coloration of live animals blackish-brown (Fig. 1A) with a pattern of contrasting light brown paraterga and posterior halves of midbody metaterga and epiproct, dark brown to light brown head, legs and antennae; coloration in alcohol faded after two months of preservation, paraterga, legs and epiproct being light brown to whitish; head to metazonae 3 blackish, thereafter metazonae with a light brown to whitish cross (Fig. 1B, D, F); venter and a few basal podomeres light brown to yellow-brown, legs increasingly darker brown distally (Fig. 1B–J).

Clypeolabral region and vertex sparsely setose, epicranial suture distinct. Antennae moderately long (Fig. 1A–C), reaching body segment 3 (♂) or 2 (♀) when stretched dorsally. In width, head < segment 3 = 4 < collum < 2 < 5–17 (♂, ♀); thereafter body gently and gradually tapering. Collum with three transverse rows of setae: 4+4 anterior, 3+3 intermediate and 5+5 posterior; a small incision laterally in posterior half; caudal corner of paraterga rounded, slightly declined ventrad, produced behind rear tergal margin (Fig. 1B, C).

Tegument leathery and shining, prozonae very finely shagreened, metaterga leathery, finely microgranulate and delicately rugulose; surface below paraterga finely microgranulate. Postcollum metaterga with two transverse rows of setae on evident oblong ridges: 2+2 in anterior (pre-sulcus) and 3+3 in posterior (post-sulcus) row, caudal row being more strongly developed than anterior one (Fig. 1B–F, H); behind segment 10, metaterga with: 2+2 in anterior and 3(4)+3(4) in posterior row. Tergal setae long, strong, slender, about 1/3 of metatergal length. Axial line visible on metaterga. Paraterga very strongly developed (Fig. 1B–F, H), especially well so in ♂, set at about 1/4 midbody height, mostly upturned, all lying high, but always below dorsum; shoulders well-developed, mostly rounded; caudal corner almost completely to fully pointed, extending increasingly beyond tergal margin, posterior edge mostly oblique, especially strongly so on segments 16–19 (Fig. 1F, H); paraterga very thin blunt blades in lateral view, a little thicker only on pore-bearing segments. Calluses on paraterga delimited by a sulcus only dorsally. Paraterga 2 broad, anterior edge angular, lateral edge with two evident incisions in anterior half; posterior edge slightly concave (Fig. 1B, C). Lateral edge of paraterga with evident incisions, one in anterior 1/3, the other at midway, caudal incision being smaller in pore-bearing segments. Ozopores evident, lateral, lying in an ovoid groove at about 1/3 in front of caudal corner. Transverse sulcus usually distinct (Fig. 1B, D, F), slightly incomplete on segment 19, complete on metaterga 4–18, deep, reaching bases of paraterga, clearly beaded at bottom. Stricture between pro- and metazonae wide, clearly ribbed at bottom down to base of paraterga (Fig. 1B–E). Pleurosternal carinae complete crests with a sharp caudal tooth on segment 7 (♂, ♀), a small, caudal, mostly sharp tooth until segment 17 (♂) or 16 (♀), thereafter missing (Fig. 1C, E, H). Epiproct (Fig. 1F, G) conical, flattened dorsoventrally, with two strong apical papillae; tip subtruncate; pre-apical papillae evident, lying close to tip. Hypoproct roundly subtrapeziform (Fig. 1G), setiferous knobs at caudal edge well-separated and evident.

**Figure 2.**
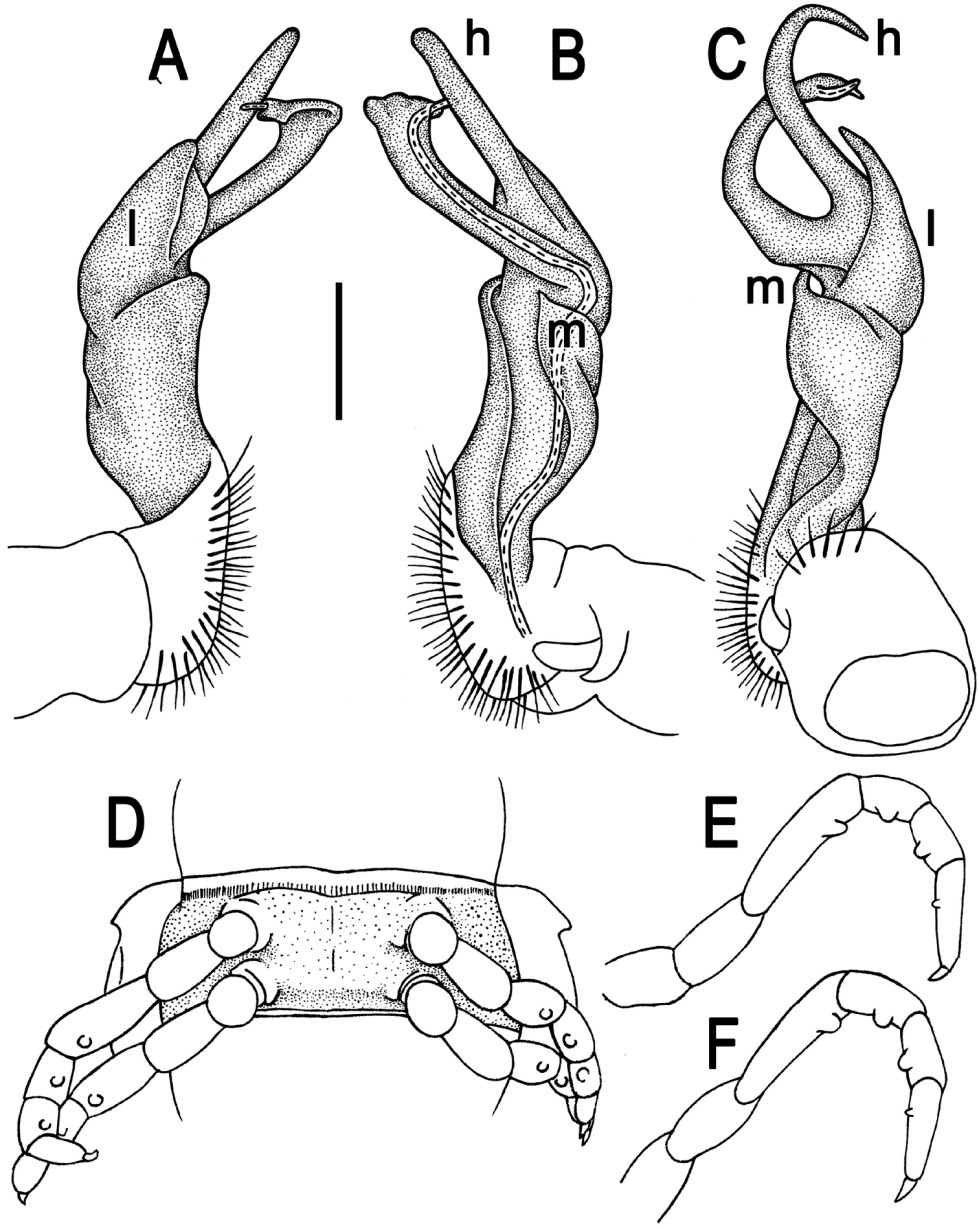
*Tylopus corrugatus* sp. n., ♂ holotype; **A–C** right gonopod, lateral, mesal and anteromesal views, respectively, scale bar: 0.2 mm **D** sternum of segment 10. **E, F** leg of segment 10, depicted not to scale.

Sterna very densely setose, with a small cone caudally near each coxa, rear cones being a bit better developed than front ones (Fig. 2D); a deeply notched sternal lobe between ♂ coxae 4 (Fig. 1I, J). Legs moderately long and slender, midbody ones ca 1.0–1.2 (♂) or 0.9–1.1 times (♀) as long as body height, ♂ legs of segments 5–16 with an evident adenostyle (tubercle) on femur, postfemur, tibia and tarsus (Fig. 2D–F); tarsal brushes present until ♂ legs 7.

**Figure 3.**
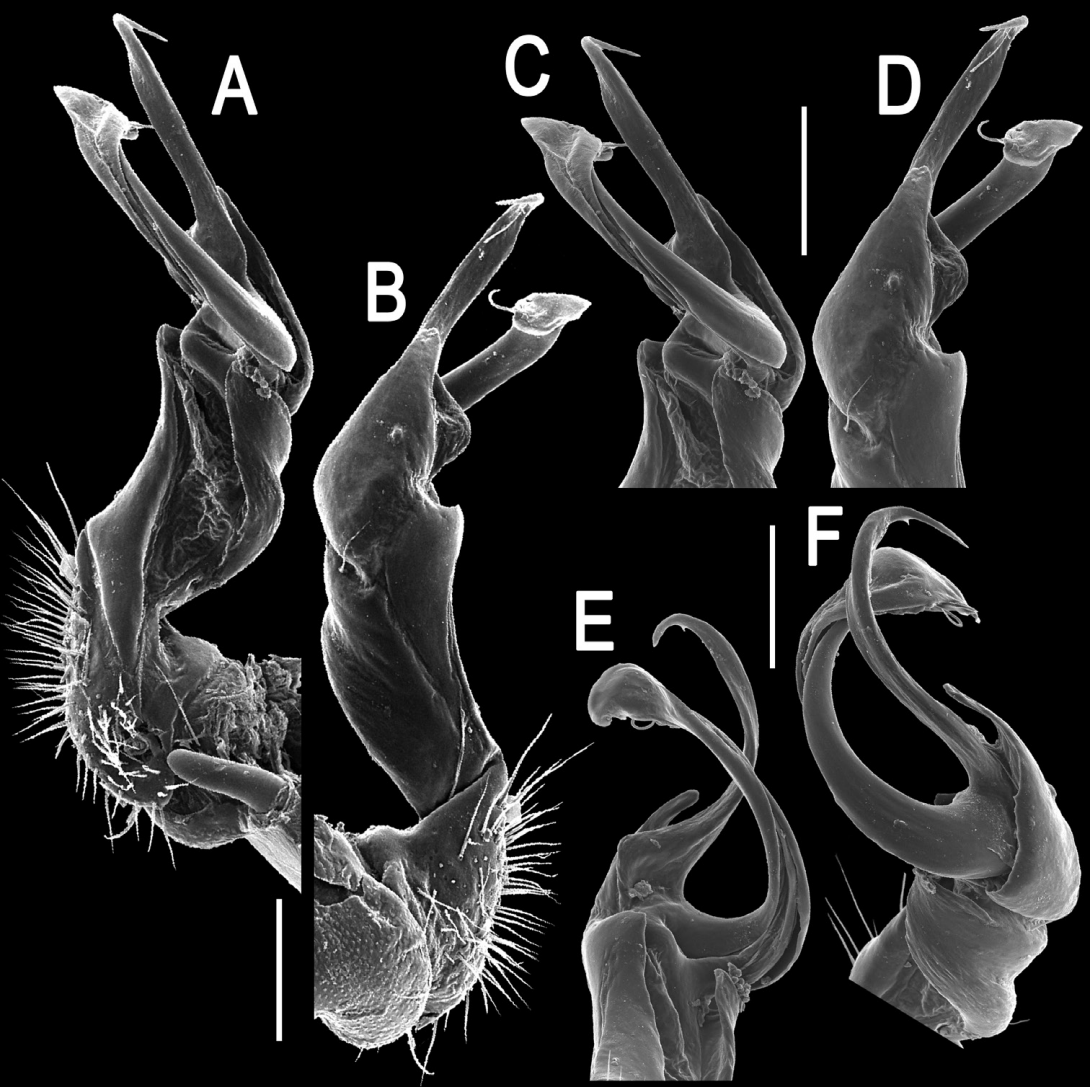
*Tylopus corrugatus* sp. n., ♂ paratype, right gonopod; **A, B** mesal and lateral views, respectively **C–F** distal part, mesal, lateral, posterior and anterior views, respectively. Scale bars: 0.2 mm.

Gonopods (Figs 2A–C, 3) simple; coxa a little curved caudad, sparsely setose distoventrally. Prefemur densely setose, about 1/3 as long as femorite + “postfemoral” part. Femorite rather stout, expanded distad, slightly curved, showing a mesal groove; lobe **l** simple; process **m** apicoventral and spiniform; solenophore long and slender, typically coiled, tip subtruncate; process **h** strongly developed, curved and acute, longer than solenophore.

#### Tylopus parahilaroides sp. n.

Taxon classificationAnimaliaPolydesmidaParadoxosomatidae

http://zoobank.org/0E564999-E51A-4D50-9099-8857FF80E9D6

Figs 4 , 5

##### Holotype.

♂ (CUMZ), Thailand, Loei Province, Phuluang District, Phuluang Wildlife Sanctuary, 1,486 m a.s.l., 17°16'44.9"N, 101°31'10.2"E, 20.07.2011, leg. Sira Noommeechai.

##### Paratype.

1 ♀ (CUMZ), same data, together with holotype.

##### Name.

To emphasize the close resemblance to *Tylopus hilaroides* Golovatch, 1984.

##### Diagnosis.

Very similar to *Tylopus hilaroides*, especially as regards its gonopod conformation, but differs in the presence of two rows of setae on metaterga 3–18 (an anterior transverse row of 2+2 setae and a posterior row of 4+4 insertion points versus solely an anterior transverse row of 2+2 setae), by the transverse sulcus visible starting already from metatergum 4 (versus metatergum 5), as well as in gonopod process **z** with two evident spines along distal margin (versus three spines) and process **h** being smaller (versus stouter).

##### Description.

Length 34 (♂) or 33 mm (♀), width of midbody pro- and metazonae 3.1 and 4.3 mm (♂) or 3.2 and 4.1 mm (♀), respectively.

**Figure 4.**
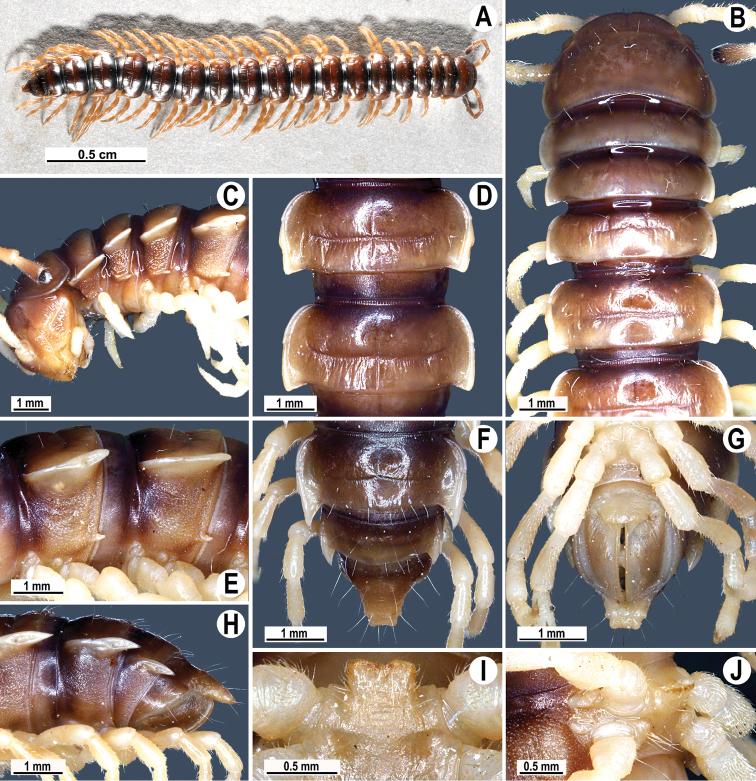
*Tylopus parahilaroides* sp. n., ♂ holotype; **A** habitus, live coloration **B, C** anterior part of body, dorsal and lateral views, respectively **D, E** segments 10 and 11, dorsal and lateral views, respectively **F–H** posterior part of body, dorsal, ventral and lateral views, respectively **I, J** sternal cones between coxae 4, caudal and lateral views, respectively.

Coloration of live animals dark castaneous brown (Fig. 4A); legs red-brown, venter and a few basal podomeres light brown to yellow-brown; coloration of alcohol material after a half year preservation faded to dark brown; antennae and epiproct light brown to pallid, venter and a few basal podomeres light brown to pallid (Fig. 4B–H).

Clypeolabral region and vertex sparsely setose, epicranial suture distinct. Antennae moderately long (Fig. 4A), reaching behind body segment 3 (♂, ♀) when stretched dorsally. In width, head < segment 3 < 4 < 5 < collum < segment 2 < 6–17 (♂) or head < segment 3 < 4 < collum < segment 2 < 5–17 (♀); thereafter body gently and gradually tapering. Collum with three transverse rows of setae: 4+4 anterior, 3+3 intermediate, and 4+4 posterior; a setigerous incision laterally in posterior 1/3; caudal corner of paraterga very narrowly rounded, not drawn behind rear tergal margin (Fig. 4B, C).

Tegument rather smooth and shining, prozonae finely shagreened, metaterga often rugose (Fig. 4A–F); surface below paraterga finely microgranular (Fig. 4C, E, H). Postcollum metaterga with an anterior transverse row of 2+2 setae visible at least as insertion points, and a posterior row of 4+4 insertion points. Tergal setae long, strong, slender, about 1/3 of metatergal length. Axial line clearly visible both on pro- and metazonae. Paraterga strongly developed (Fig. 4B–H), especially so in ♂, lying rather high (at 1/3 of midbody height), slightly upturned, but lying below dorsum; anterior edge rounded, caudal corner very narrowly rounded, starting from segment 13 extending increasingly beyond rear tergal margin, pointed, on segments 15–19 tips strongly curved mesad (Fig. 4F, H); lateral edge on poreless segments with two evident (anterior larger, posterior one smaller) setigerous incisions in anterior 1/3, but with only one strong (anterior) incision on pore-bearing segments (Fig. 4B, D, F); posterior edge oblique. Calluses on paraterga narrow, delimited by a sulcus only dorsally in segments 2–3, but both dorsally and ventrally in following segments. Paraterga 2 broad, posterior edge clearly oblique. Paraterga 2 and 3 broadly angular anteriorly, following segments with rounded anterior edges (Fig. 4B). Ozopores evident, lateral, lying in an ovoid groove at about 1/3 in front of posterior edge of metaterga. Transverse sulcus usually distinct (Fig. 4B, D, H), slightly incomplete on segments 4 and 19, complete on metaterga 5–18 (♂, ♀), narrow, wavy, rather deep, not reaching bases of paraterga, at most faintly ribbed at bottom. Stricture between pro- and metazonae broad and deep, beaded at bottom down to well below base of paraterga (Fig. 4B, D, H). Pleurosternal carinae complete crests with a sharp caudal tooth on segments 2–4, thereafter split into a sharp front and a sharp caudal tooth, the former gradually turning into a bulge, the latter tooth gradually reduced until segment 17 (♂, ♀). Epiproct (Fig. 4F, G) conical, flattened dorsoventrally, with two evident apical papillae; tip subtruncate; pre-apical papillae small, lying rather close to tip. Hypoproct roundly subtrapeziform, setiferous knobs at caudal edge small and well-separated (Fig. 4G).

**Figure 5.**
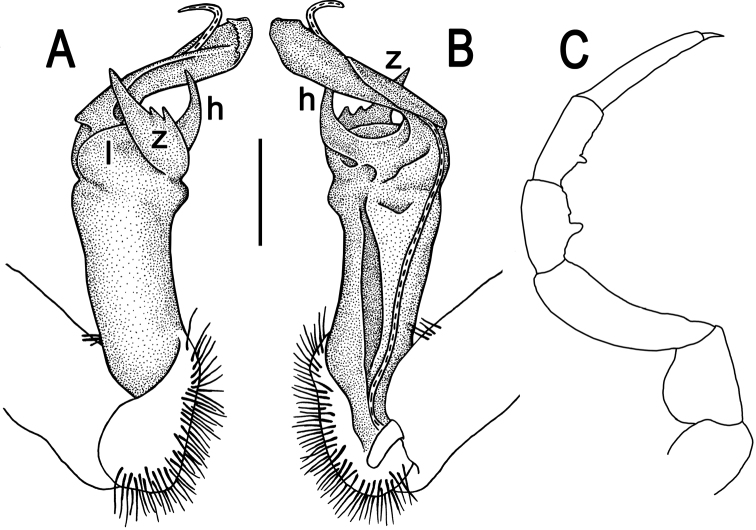
*Tylopus parahilaroides* sp. n., ♂ holotype; **A, B** right gonopod, lateral and mesal views, respectively, scale bar: 0.2 mm **C** leg of segment 10, depicted not to scale.

Sterna densely setose, without modifications; a single, linguiform, sternal lobe between ♂ coxae 4 (Fig. 4I, J). Legs rather long and slender, midbody ones ca 1.2–1.3 (♂) or 1.1–1.4 times (♀) as long as body height; appressed setation ventrally on coxa, prefemur and femur, but tarsal brushes absent; ♂ prefemora distinctly bulged laterally (Fig. 5C), ♂ postfemora and tibiae on segments 7–17 with an evident adenostyle at midway on ventral side (Fig. 5C).

Gonopods (Fig. 5A, B) simple; coxa a little curved caudad, sparsely setose distoventrally. Prefemur densely setose, about 1/3 as long as femorite + “postfemoral” part. Femorite rather stout, expanded distad, slightly curved, showing a mesal groove; lobe **l** simple; process **z** with two evident spines along dorsal margin; process **h** short and slender, curved, with an acute tip; solenophore long and slender, typically coiled, tip subtruncate.

#### Tylopus trigonum sp. n.

Taxon classificationAnimaliaPolydesmidaParadoxosomatidae

http://zoobank.org/7B4546E1-7163-433F-816D-6DCB8E010BAF

Figs 6 , 7

##### Holotype.

♂ (CUMZ), Thailand, Tak Province, Umphang District, Pa Wai Waterfall, 804 m a.s.l., 16°34'29.6"N, 98°50'3.2"E, 20.01.2011, leg. C. Sutcharit & N. Likhitrakarn.

##### Paratypes.

1 ♂ (CUMZ), same data, together with holotype. 3 ♀ (CUMZ), same district, Thee Lor Sue Waterfall, 591 m a.s.l., 15°55'38.1"N, 98°45'12.8"E, 19.01.2011, leg. N. Likhitrakarn.

##### Name.

To emphasize the light brown triangle on terga; noun in apposition.

##### Diagnosis.

This new species shows a peculiar colour pattern, much like that observed in *Tylopus schawalleri* Golovatch, 2013, but differs in gonopod process **h** being rather short and coiled (versus high and strongly twisted), as well as by the presence of a process **m** (versus absent).

##### Description.

Length 21.2–27.8 (♂) or 22.1–24.0 mm (♀), width of midbody pro- and metazona 1.97–1.83 and 2.65–2.78 mm (♂) or 2.43–2.58 and 3.05–3.14 mm (♀), respectively.

**Figure 6.**
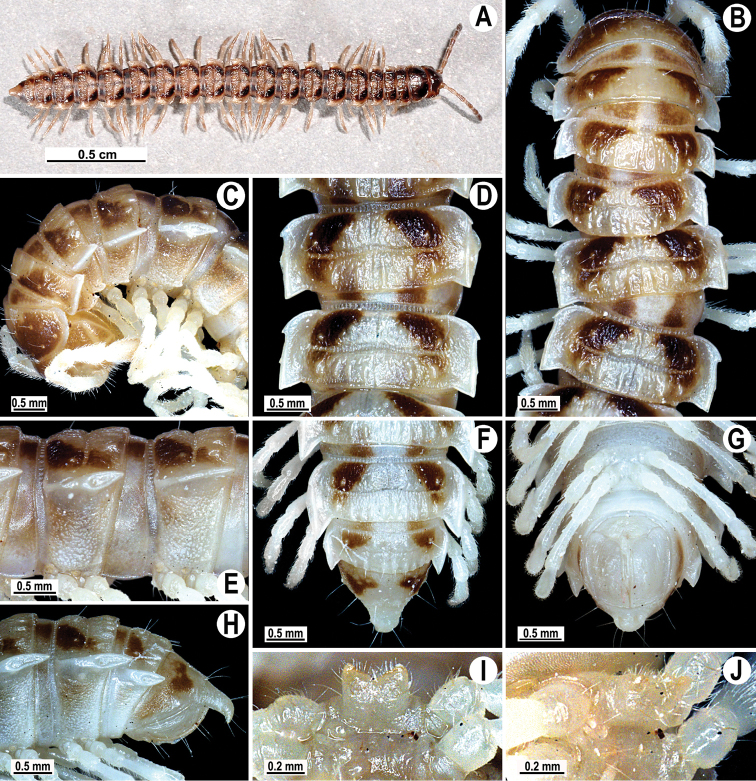
*Tylopus trigonum* sp. n., ♂ holotype; **A** habitus, live coloration **B, C** anterior part of body, dorsal and lateral views, respectively **D, E** segments 10 and 11, dorsal and lateral views, respectively **F–H** posterior part of body, dorsal, ventral and lateral views, respectively **I, J** sternal cones between coxae 4, subcaudal and sublateral views, respectively.

Coloration of live animals light brown (Fig. 6A); paraterga, legs and epiproct light brown, head and collum blackish, following terga with a light brown triangle and blackish collar covering both pro- and metazonae; coloration of alcohol material after three years of preservation faded to whitish with a pattern of a contrasting dark brown inverted triangle at anterior edge of metazonae and a light brown triangle at posterior edge of prozonae (Fig. 6B–H).

Clypeolabral region and vertex sparsely setose, epicranial suture distinct. Antennae very short (Fig. 6A, B), reaching only behind body segment 2 (♂) or collum (♀) when stretched dorsally. In width, head < segment 3 < 4 < collum < segment 2 < 5–17 (♂, ♀); thereafter body gently and gradually tapering. Collum with three transverse rows of strong setae: 4+4 anterior, 2+2 intermediate, and 4+4 posterior; a rounded incision laterally in posterior half; caudal corner of paraterga rounded, slightly declined ventrad, produced behind rear tergal margin (Fig. 6B, C).

Tegument rather smooth and shining, prozonae very finely shagreened, metaterga smooth and finely rugulose, leathery; surface below paraterga finely microgranulate (Fig. 6B–H). Postcollum metaterga with two transverse rows of setae on small knobs to oblong ridges: 2+2 in anterior (pre-sulcus), 3+3 in posterior (post-sulcus) row, caudal row more strongly developed than anterior one, starting from metaterga 11 with 2+2 in anterior and 4(3)+4(3) in posterior row. Tergal setae long, strong, slender, about 1/3 of metatergal length. Axial line visible. Paraterga very strongly developed (Fig. 6B–F, H), especially well in ♂, set high, at about 1/3 of midbody height, slightly upturned, always lying high, but below dorsum; shoulders well-developed, mostly regularly rounded and narrowly bordered, fused to callus; caudal corner narrowly rounded to fully pointed, extending increasingly beyond rear tergal margin, posterior edge clearly oblique (Fig. 6B, D, F); paraterga very thin blunt blades in lateral view, a little thicker only on pore-bearing segments. Calluses on paraterga delimited by a sulcus both dorsally and ventrally. Paraterga 2 broad, anterior edge angular, lateral edge with three evident incisions in anterior half (Fig. 6B, C). Lateral edge of following paraterga with two clear incisions, one in anterior 1/3, the other at midway, front one being particularly evident. Ozopores evident, lateral, lying in an ovoid groove at about 1/3 in front of caudal corner. Transverse sulcus usually distinct (Fig. 6B, D, H), slightly incomplete on segments 4 and 19, complete on metaterga 5–18, deep, reaching bases of paraterga, clearly ribbed at bottom. Stricture between pro- and metazonae very wide, clearly beaded at bottom down to base of paraterga (Fig. 6B, D, H). Pleurosternal carinae complete crests with a sharp caudal tooth on segment 12 (♂) or 7 (♀), thereafter increasingly well reduced in size and sharpness until segment 17 (♂) or 14 (♀), onward missing (Fig. 6C, E, H). Epiproct (Fig. 6F–H) conical, flattened dorsoventrally, subtruncate, with two evident apical papillae directed caudally, both pointed at tip; pre-apical papillae evident, lying close to tip. Hypoproct roundly subtrapeziform (Fig. 6G), small setiferous knobs at caudal edge well-separated and evident.

**Figure 7.**
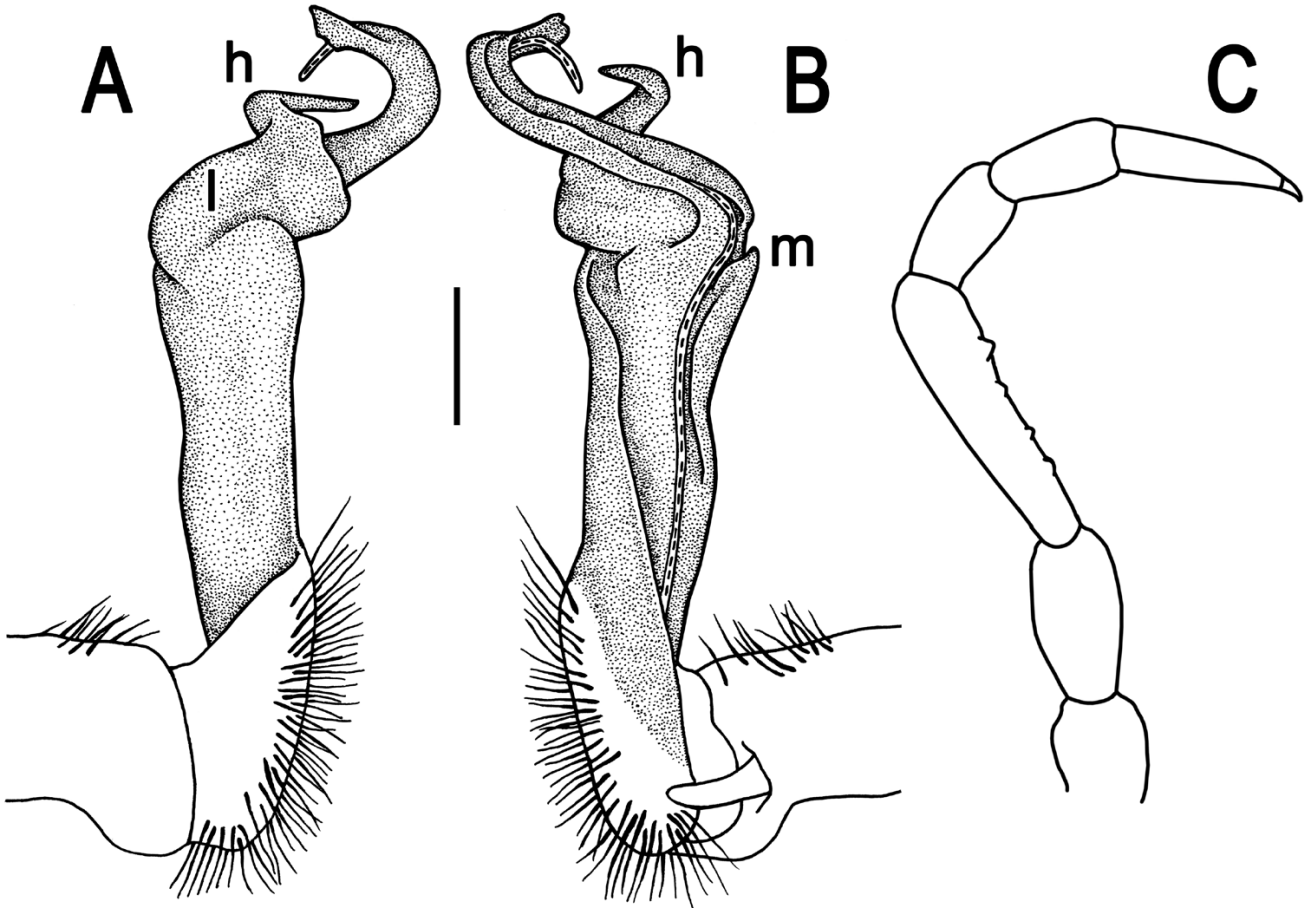
*Tylopus trigonum* sp. n., ♂ holotype; **A, B** right gonopod, lateral and mesal views, respectively, scale bar: 0.2 mm **C** leg of segment 10, depicted not to scale.

Sterna very densely setose, with a small cone caudally near each coxa; a single, linguiform, deeply medially notched sternal lobe between ♂ coxae 4 (Fig. 9I, J). Legs moderately long and slender, midbody ones ca 1.1–1.2 (♂) or 1.0–1.1 times (♀) as long as body height, ♂ femora with 2–4 small adenostyles on ventral side (Fig. 7C); tarsal brushes present until ♂ leg 7.

Gonopods (Fig. 7A, B) very simple; coxa a little curved caudad, sparsely setose distoventrally. Prefemur densely setose, about 1/3 as long as femorite + “postfemoral” part. Femorite rather slender, expanded distad, slightly curved, showing a mesal groove; lobe **l** simple; solenophore long and slender, typically coiled, tip subtruncate; process **m** evident, but not spiniform; process **h** prominent, coiled, acute at tip.

##### Remark.

The ♂ paratype on metatergum 19 shows 3+3 and 6+6 setae without knobs in the anterior and posterior rows, respectively.

#### Tylopus nodulipes

Taxon classificationAnimaliaPolydesmidaParadoxosomatidae

(Attems, 1953)

Figs 8 , 9

Agnesia nodulipes Attems, 1953: 174 (D).
Agnesia nodulipes – Jeekel 1965: 98 (R).
Tylopus nodulipes – Jeekel 1968: 60 (M); Hoffman 1973: 371(M, D); Golovatch 1983: 182 (M); 1984: 69 (M, D); Golovatch and Enghoff 1993: 90 (M, D); Enghoff et al. 2004: 40 (R); Likhitrakarn et al. 2010: 25 (R, D); Nguyen 2012: 301 (R, D).

##### Lectotype

♂ (here designated) of *Agnesia nodulipes* (NHMW-3986), Laos, Luang Prabang, 1938–1939, leg. C. Dawydoff.

Lectotype designation proposed herewith is necessary to ensure the species is based on a complete ♂ coming from a certain locality, because (1) Attems (1953) provided no information on the number and sex of syntypes, and (2) he stated their provenance to have been both from Luang Prabang, Laos and Mount Fan-Si-Pan, Lao Cai Province, Vietnam. No paralectotype material could be traced in the Vienna Museum.

##### Redescription.

**Figure 8.**
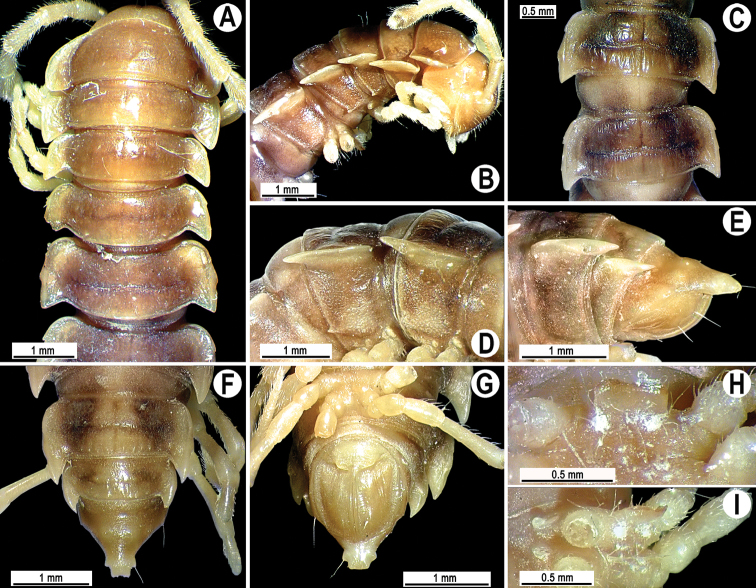
*Tylopus nodulipes* (Attems, 1953), ♂ lectotype; **A, B** anterior part of body, dorsal and lateral views, respectively **C** segments 10 and 11, dorsal view **D** segments 9–11, lateral view **E–G** posterior part of body, lateral, dorsal and ventral views, respectively **H, I** sternal cones between coxae 4, caudal and lateral views, respectively.

Lectotype ca 24 mm long, width of midbody pro- and metazonae 2.1 and 2.9 mm (vs 3.0 in width, as given in the available description (Attems 1953)). Coloration of alcohol material after long preservation rather uniformly light reddish brown (Fig. 8A–G) with light yellow antennae, paraterga, epiproct and legs (versus dark maroon with light yellowish brown mid-dorsal parts of prozonae, paraterga a little lighter, antennae light chestnut brown and legs yellow brown, as given in the original description (Attems 1953)).

Clypeolabral region densely setose; vertex rather smooth, only faintly rugulose; epicranial suture distinct. Antennae rather long and slender (Fig. 8A, B), reaching behind body segment 3 when stretched dorsally. In width, head < segments 3 and 4 < collum < 2 < 5–16, gently and gradually tapering thereafter. Collum smooth, with three transverse rows of setae, 4+4 anterior, 2+2 intermediate, and 4+4 posterior; caudal corner of paraterga subrectangular, narrowly rounded (Fig. 8A, B), drawn behind rear tergal margin.

Tegument smooth and shining; metaterga rugulose, prozonae finely shagreened, surface below paraterga finely microgranulate. Metaterga 2–17 with two transverse rows of setae: 2+2 in anterior (pre-sulcus) row and 3(2)+3(2) in posterior (post-sulcus) one, setae being borne on very small tubercles growing a little larger laterally, metaterga 18 and 19 with two transverse rows of setae: 2+2 in anterior and 4+4 in posterior row. Tergal setae long, strong, slender, about 1/3 of metatergal length. Axial line visible. Paraterga very strongly developed (Fig. 8A–G), subhorizontal, lying below dorsum, thin blunt blades in lateral view, a little thicker only on pore-bearing segments, on postcollum segments extending increasingly beyond rear tergal margin, nearly pointed to pointed, caudal tip on paraterga 18–19 clearly curved mesad. Calluses delimited by a sulcus only dorsally, rather narrow. Paraterga 2 broad, slightly upturned, anterior edge rounded, lateral edge with three small incisions in anterior half; posterior edge oblique (Fig. 8A, B). Anterior edge of postcollum metaterga broadly rounded, bordered and fused to callus, lateral edge with two small incisions in anterior half on poreless segments, with only one incision near front 1/3 on pore-bearing ones. Ozopores evident, lateral, lying inside an ovoid groove at about 1/3 of metazonital length. Transverse sulcus complete on metaterga 5–18, incomplete on metatergum 19, rather wide, reaching bases of paraterga, faintly beaded at bottom (Fig. 8A, C, F). Stricture between pro- and metazonae very wide, shallow, faintly beaded at bottom down to base of paraterga. Pleurosternal carinae complete crests only on segment 2 (Fig. 8B), with anteriorly bulged crests and a sharp denticle caudally on segments 3–8, thereafter only a small sharp caudal tooth on segments 9–15, onward missing (Fig. 8B & D). Epiproct (Fig. 8F, G) conical, flattened dorsoventrally, apical papillae evident; tip subtruncate; pre-apical papillae rather large, lying close to tip. Hypoproct (Fig. 8G) roundly subtrapeziform, setigerous knobs at caudal margin evident and well-separated.

**Figure 9.**
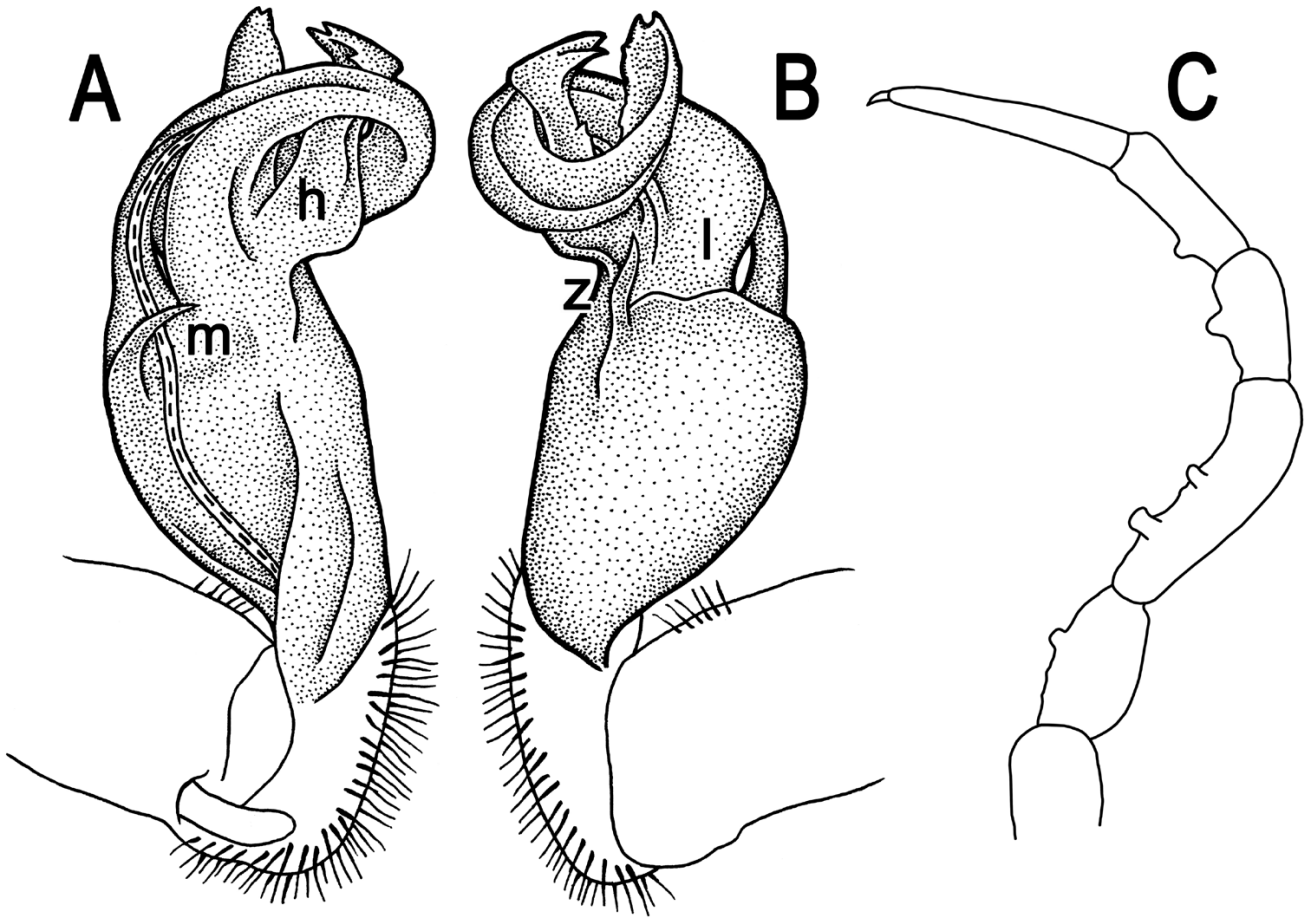
*Tylopus nodulipes* (Attems, 1953), ♂ lectotype; **A, B** left gonopod, mesal and lateral views, respectively **C** leg of segment 10, depicted not to scale.

Sterna sparsely setose, starting from segment 6 with a small cone caudally near each coxa, rear cones being a little larger than front ones; a rather large, linguiform, densely setose, sternal lobe between ♂ coxae 4 (Fig. 8H, I). Legs moderately long and slender, midbody ones ca 1.2–1.3 times as long as body height, legs of segments 8–18 with an evident adenostyle on each prefemur, postfemur and tibia, with two adenostyles on each femur (Fig. 9C); tarsal brushes present only until ♂ legs 4.

Gonopods (Fig. 9A, B) rather simple; prefemur densely setose, about 1/3 as long as femorite + “postfemoral” part. Femorite stout, expanded distad, slightly curved, showing a mesal groove; lobe **l** simple; solenophore long and slender, typically coiled, tip subtruncate; process **h** high, strongly twisted, tip bifid; process **m** rather long and spiniform, process **z** knife-shaped.

##### Remarks.

This is the type species of *Tylopus* Jeekel, 1968, originally recorded from two localities: Luang Prabang Province, Laos and Mount Fan-Si-Pan, Lao Cai Province, Vietnam (Attems 1953). Golovatch (1984) redescribed and illustrated only a gonopod, but the locality remained unclear. So the lectotype is herewith selected for the sole type specimen still kept in the Vienna Museum.

This species has recently been reported from Nam Xay Commune (22°05’N, 104°05’E), 1,000 m a.s.l., Van Ban District, Lao Cai Province; Son Tay Commune, 600 m a.s.l., Huong Son District, Ha Tinh Province; and Chem Waterfall, 430 m a.s.l., Pu Mat National Park (18°46'–19°12'N, 104°01'–104°56'E), Nghe An Province, Vietnam (Nguyen 2012).

#### Tylopus hilaris

Taxon classificationAnimaliaPolydesmidaParadoxosomatidae

(Attems, 1937)

Figs 10 , 11

Anoplodesmus hilaris Attems, 1937: 105 (D).
Anoplodesmus hilaris – Attems 1938: 215 (D).
Agnesia hilaris – Jeekel 1965: 97 (M, D).
Tylopus hilaris – Jeekel 1968: 60 (M); Hoffman 1973: 371 (M, D); Golovatch 1983: 182 (M); 1984: 69 (M, D); Golovatch and Enghoff 1993: 90 (M, D); Enghoff et al. 2004: 40 (R); Likhitrakarn et al. 2010: 25 (R, D); Nguyen 2012: 301 (R, D).

##### Holotype

♂ of *Anoplodesmus hilaris* (NHMW-4248), Vietnam, Danang Prov., Mount Bana, 1,500 m, 28.09.1931, leg. C. Dawydoff.

##### Redescription.

**Figure 10.**
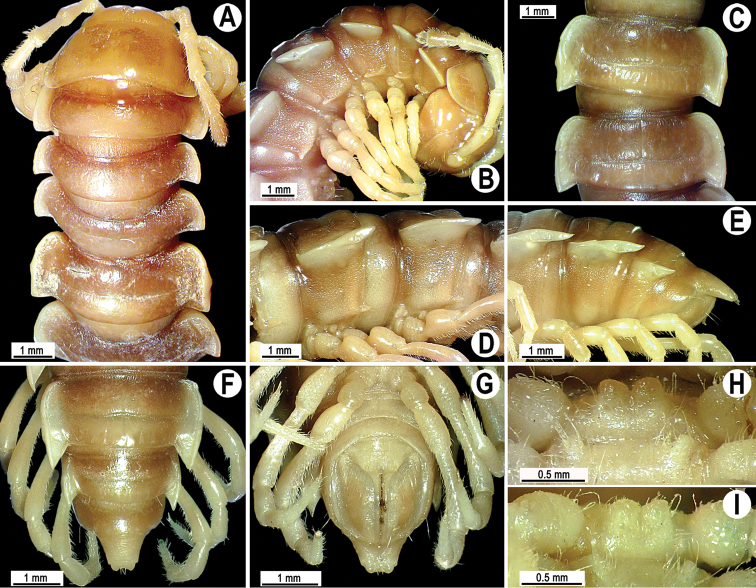
*Tylopus hilaris* (Attems, 1937), ♂ holotype; **A, B** anterior part of body, dorsal and lateral views, respectively **C** segments 10 and 11, dorsal view **D** segments 9–11, lateral view **E–G** posterior part of body, lateral, dorsal and ventral views, respectively **H, I** sternal cones between coxae 4, subcaudal and sublateral views, respectively.

Length ca 38 mm, width of midbody pro- and metazonae 3.7 and 5.1 mm, respectively (vs 3.4 and 5.0 mm in width, as given in the available descriptions (Attems 1937, 1938)). Coloration of alcohol material after long preservation brown (Fig. 10A–G) with light yellow antennae, paraterga, epiproct and legs (versus dark brown with prozonae and posterior halves of metazonae blackish brown; edge of paraterga, antennae and legs yellowish brown, as given in the descriptions (Attems 1937, 1938)).

Clypeolabral region densely setose, vertex smooth, epicranial suture distinct. Antennae rather long (Fig. 10A, B), reaching behind body segment 3 when stretched dorsally. In width, head < segments 3 and 4 < collum < 2 < 5–16, gently and gradually tapering thereafter. Collum smooth, with three transverse rows of setae, 4+4 anterior, 2+2 intermediate, and 3+3 posterior; caudal corner of paraterga subrectangular, narrowly rounded (Fig. 10A, B). Tegument smooth and shining; metaterga faintly rugulose, prozonae finely shagreened, surface below paraterga finely microgranulate. Postcollum metaterga with an anterior transverse row (pre-sulcus) of 2+2, mostly abraded setae; caudal (post-sulcus) row barely traceable as 3+3 insertion points. Tergal setae short, simple, slender, about 1/5 metatergal length. Axial line barely visible, starting from collum. Paraterga very strongly developed (Fig. 10A–F), all subhorizontal and lying below dorsum, thin blunt blades in lateral view, a little thicker only on pore-bearing segments, on postcollum segments extending increasingly beyond rear tergal margin, nearly pointed to pointed, caudal tips on paraterga 17–19 evidently curved mesad. Calluses delimited by a sulcus only dorsally, rather narrow. Paraterga 2 broad, anterior edge rounded, lateral edge with three small incisions in anterior half; posterior edge concave (Fig. 10A). Anterior edge of postcollum segments broadly rounded, bordered and fused to callus, lateral edge with two small incisions in anterior half on poreless segments, with only one incision near front 1/3 on pore-bearing ones; posterior edge oblique. Ozopores evident, lateral, lying inside an ovoid groove at about 1/4 metazonital length in front of caudal corner. Transverse sulcus complete on metaterga 5–18, incomplete on metaterga 4 and 19, rather deep, wide, line-shaped, reaching bases of paraterga, ribbed at bottom (Fig. 10A, C, F). Stricture between pro- and metazonae shallow, broad, beaded at bottom down to base of paraterga. Pleurosternal carinae complete crests only on segment 2 (Fig. 10B), with a sharp tooth caudally on segments 3–7, only a small sharp caudal tooth on segments 8–16, onward missing (Fig. 10A, D, E). Epiproct (Fig. 10E–G) conical, flattened dorsoventrally, apical papillae very small; tip subtruncate; pre-apical papillae very small, lying close to tip. Hypoproct (Fig. 10G) roundly subtriangular, setiferous knobs at caudal margin small and well-separated.

**Figure 11.**
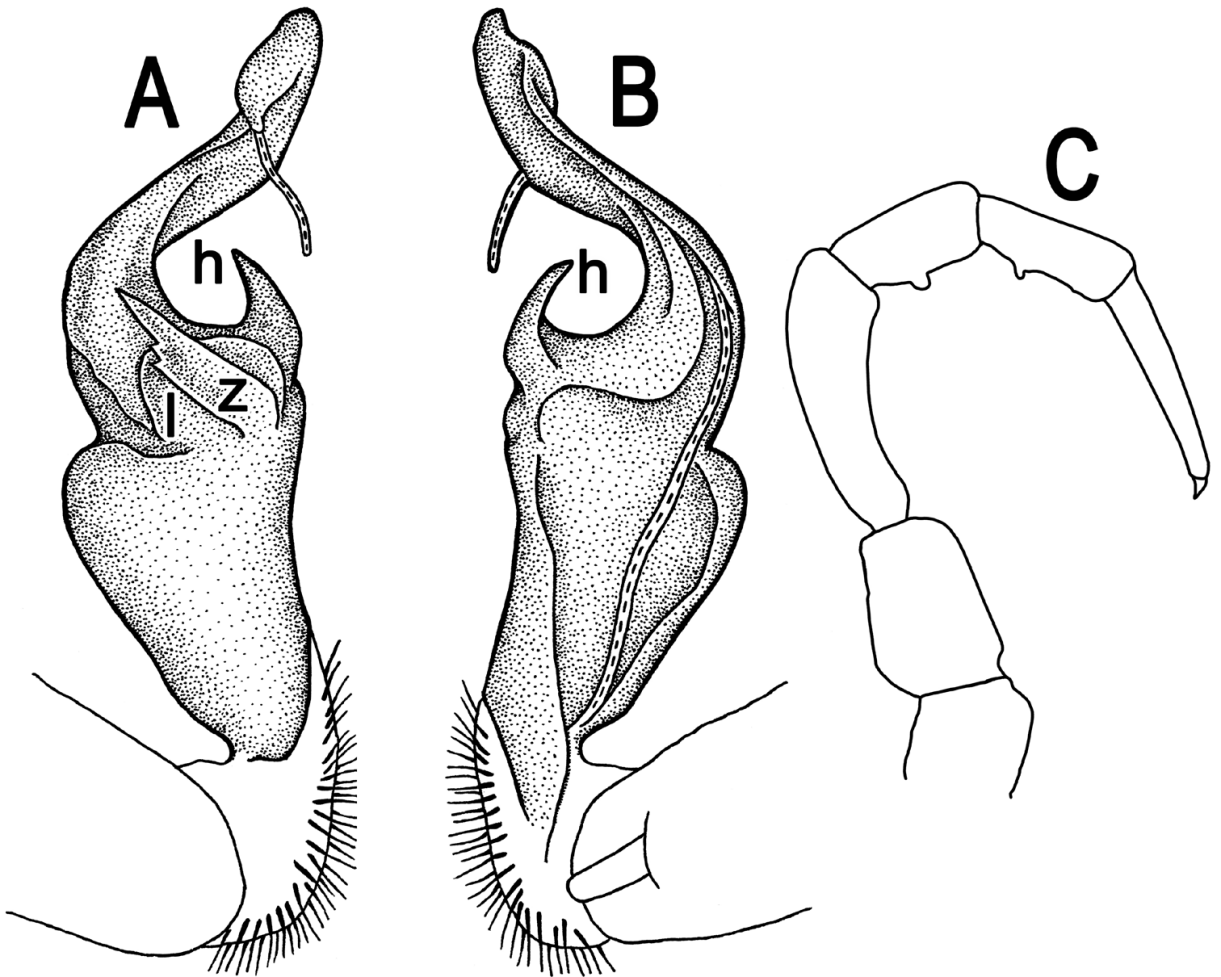
*Tylopus hilaris* (Attems, 1937), ♂ holotype; **A, B** right gonopod, lateral and mesal views, respectively **C** leg of segment 10, depicted not to scale.

Sterna sparsely setose, until segment 6 with an evident cone caudally near coxae; on segment 4 with an evident, central cone between coxae; on segment 5 with a small central lobe with a paramedian pair of evident, sparsely setose, apical cones between coxae (Fig. 10H, I). Legs long and slender, midbody ones ca 1.2–1.3 (♂) or 0.8–0.9 (♀) times as long as midbody height, all legs until segment 17 with an evident adenostyle on each postfemur and tibia (Fig. 11C); tarsal brushes absent.

Gonopods (Fig. 11A, B) simple; coxa a little curved caudad, sparsely setose distoventrally. Prefemur densely setose, about 1/3 as long as femorite + “postfemoral” part. Femorite rather stout, expanded distad, slightly curved, showing a mesal groove; lobe **l** simple; process **z** with two small spines along ventral margin; process **h** short and stout, curved, tip acute; solenophore long and slender, typically coiled, tip subtruncate.

##### Remark.

Endemic to Vietnam, *Tylopus hilaris* is currently known from Mount Bana, 1,500 m a.s.l., Danang Province (Attems, 1937); Bach Ma National Park (16°05'–16°05'N, 107°43'–107°53'E), Thua Thein Hue Province; Mount Ngoc Linh (15°00'–15°18'N, 107°41'–108°01'E), Kon Tum Province, central Vietnam (Nguyen 2012).

#### Tylopus sigma

Taxon classificationAnimaliaPolydesmidaParadoxosomatidae

(Attems, 1953)

Figs 12 –13

Sundanina sigma Attems, 1953: 171 (D).
Sundanina sigma – Jeekel 1968: 60 (M).
Tylopus sigma – Golovatch 1983: 182 (M); 1984: 69 (M, D); Golovatch and Enghoff 1993: 90 (M, D); Enghoff et al. 2004: 40 (R); Likhitrakarn et al. 2010: 26 (R, D).

##### Lectotype ♂

(here designated) of *Sundanina sigma* (NHMW-3987), Vietnam, Lao Cai Prov., Sapa (= Chapa), 1938–1939, leg. C. Dawydoff.

##### Paralectotypes.

2 ♂ of *Sundanina sigma* (NHMW-3987), same data, together with lectotype.

Lectotype designation proposed herewith is necessary to ensure the species is based on a complete ♂, because Attems (1953) provided no information on the number and sex of syntypes.

##### Redescription.

**Figure 12.**
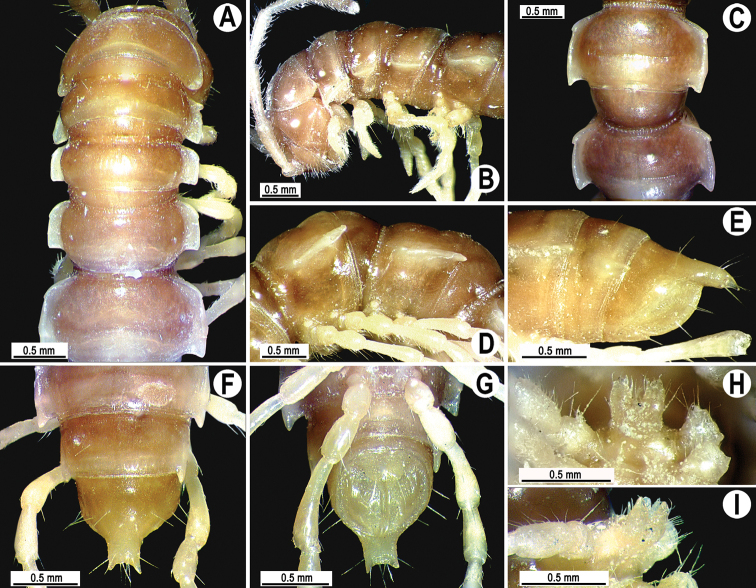
*Tylopus sigma* (Attems, 1953), ♂ paralectotype; **A, B** anterior part of body, dorsal and lateral views, respectively **C** segments 10 and 11, dorsal view **D** segments 9–11, lateral view **E–G** posterior part of body, lateral, dorsal and ventral views, respectively **H, I** sternal cones between coxae 4, caudal and lateral views, respectively.

Length 18–19 mm (♂), width of midbody pro- and metazonae 1.0–1.1 and 1.5–1.7 mm (♂), respectively (versus length 25 mm and width of midbody metazonae 1.6 mm, as given in the original description (Attems 1953)). Lectotype 18 mm long, 1.0 and 1.5 mm wide on midbody pro- and metazonae, respectively. Coloration of alcohol material after long preservation rather uniformly brown (Fig. 12A–G) (versus blackish brown with edges of paraterga and legs yellowish brown, as given in the description (Attems 1953)).

Clypeolabral region sparsely setose, vertex smooth, epicranial suture distinct. Antennae long and slender (Fig. 12B), extending behind body segment 5 (♂) dorsally. In width, head < segments 2–4 < collum < 5–17 (♂), gently and gradually tapering thereafter. Collum smooth, with three transverse rows of setae, 4+4 anterior, 2+2 intermediate, and 3+3 posterior; caudal corner of paraterga subrectangular, narrowly rounded (Fig. 12A). Tegument smooth and shining; metaterga and prozonae finely shagreened; surface below paraterga finely microgranulate. Postcollum with an anterior (pre-sulcus) transverse row of 2+2, mostly abraded setae; caudal (post-sulcus) row barely traceable as 3+3 insertion points. Tergal setae short, simple, slender, about 1/3 of metatergal length. Axial line barely visible, starting from collum. Paraterga very strongly developed (Fig. 12A, C, F, G), mostly subhorizontal and lying below dorsum, thin blunt blades in lateral view, a little thicker only on pore-bearing segments, posterior edge concave, caudal tip narrowly rounded. Calluses delimited by a sulcus only dorsally, rather narrow. Paraterga 2 broad, slightly upturned, anterior edge nearly straight, lateral edge with three more or less evident incisions; posterior edge clearly concave (Fig. 12A, B). Anterior edge of postcollum segments oblique, bordered and fused to callus, lateral edge with a strong incision near front 1/3; posterior edge oblique. Ozopores evident, lateral, lying inside an ovoid groove at about 1/4 metazonital length before caudal corner. Transverse sulcus complete on metaterga 5–18, shallow, not reaching bases of paraterga, faintly beaded at bottom (Fig. 12A, C, F). Stricture between pro- and metazonae broad, shallow, ribbed at bottom down to base of paraterga. Pleurosternal carinae complete crests only on segment 2, with a small sharp caudal tooth on segments 3–7, onward missing (Fig. 12B, D, E). Epiproct (Fig. 12E–G) conical, flattened dorsoventrally, apical papillae evident and large; tip subtruncate; pre-apical papillae small, but visible, lying close to tip. Hypoproct (Fig. 12G) roundly subtrapeziform, setiferous knobs at caudal margin small and well-separated.

**Figure 13.**
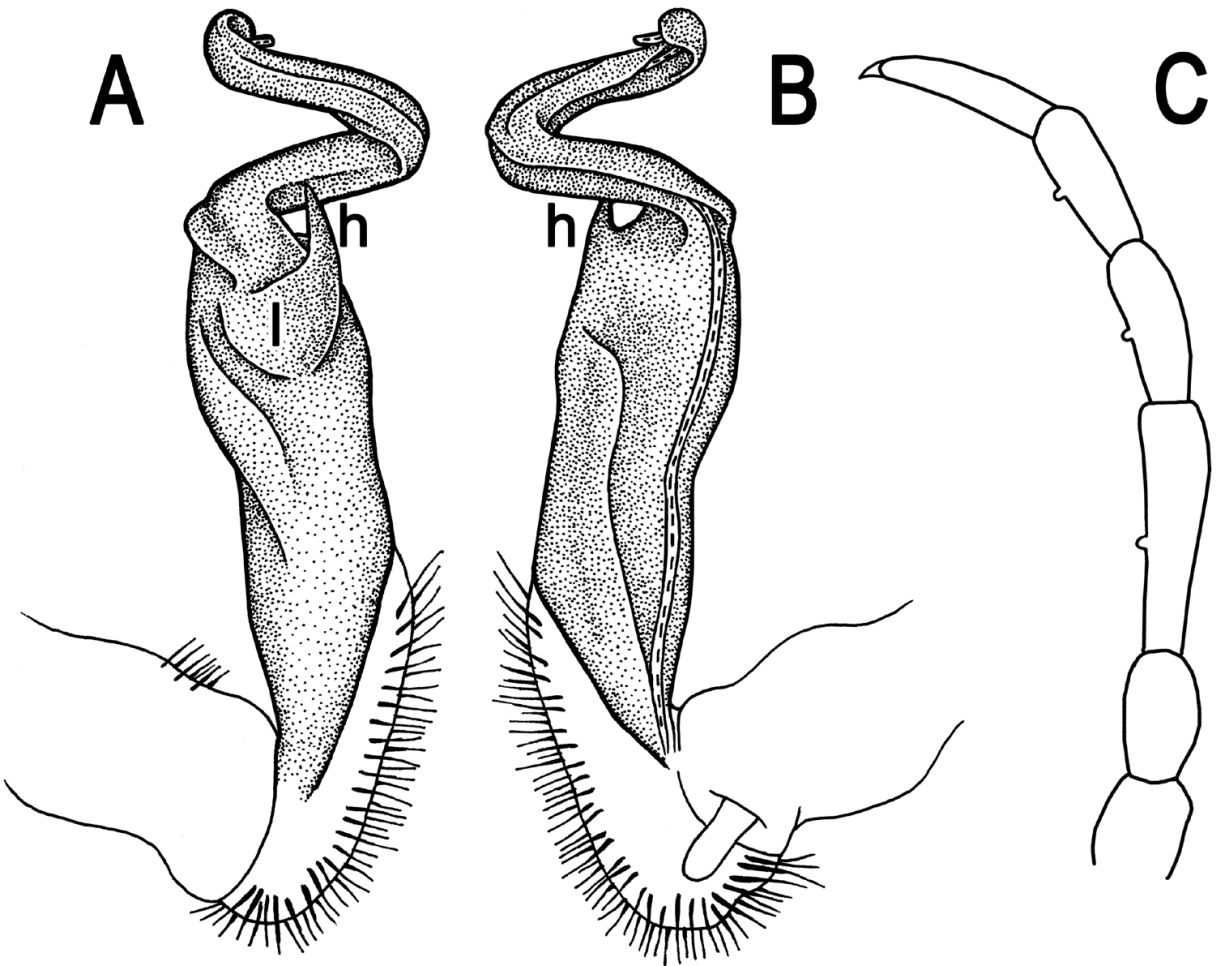
*Tylopus sigma* (Attems, 1953), ♂ lectotype; **A, B** right gonopod, lateral and mesal views, respectively **C** leg of segment 10, depicted not to scale.

Sterna sparsely setose, with a large central cone between coxae 3 and a small central lobe with a paramedian pair of evident, sparsely setose, apical cones between coxae 4 (♂) (Fig. 12H, I); segments 8–16 with a strong cone caudally near each coxa. Legs long and slender, midbody ones ca 1.4–1.5 (♂) as long as body height, legs on segments 8–18 with a small adenostyle on each prefemur, femur and postfemur (Fig. 13C); tarsal brushes absent.

Gonopods (Fig. 13A, B) very simple; coxa a little curved caudad, sparsely setose distoventrally. Prefemur densely setose, about 1/3 as long as femorite + “postfemoral” part. Femorite rather slender, expanded distad, slightly curved, showing a mesal groove; lobe **l** simple; solenophore long and slender, typically coiled, tip subtruncate; process **h** short, rather curved, tip acute.

##### Remark.

Endemic to Vietnam, *Tylopus sigma* is only known from Sapa (= Chapa), Lao Cai Province, Vietnam (Attems 1953).

#### Tylopus mutilatus

Taxon classificationAnimaliaPolydesmidaParadoxosomatidae

(Attems, 1953)

Fig. 14

Anoplodesmus mutilatus Attems, 1953: 163 (D).
Agnesia mutilata – Jeekel 1965: 98 (R).
Tylopus nodulipes – Jeekel 1968: 60 (M); Hoffman 1973: 371 (M, D); Golovatch 1983: 182 (M); 1984: 69 (M, D); Golovatch and Enghoff 1993: 90 (M, D); Enghoff et al. 2004: 40 (R); Likhitrakarn et al. 2010: 25 (R, D).

##### Syntype

♂ of *Anoplodesmus mutilatus* (NHMW-4245), locality unknown; a slide with mounted gonopod.

**Figure 14.**
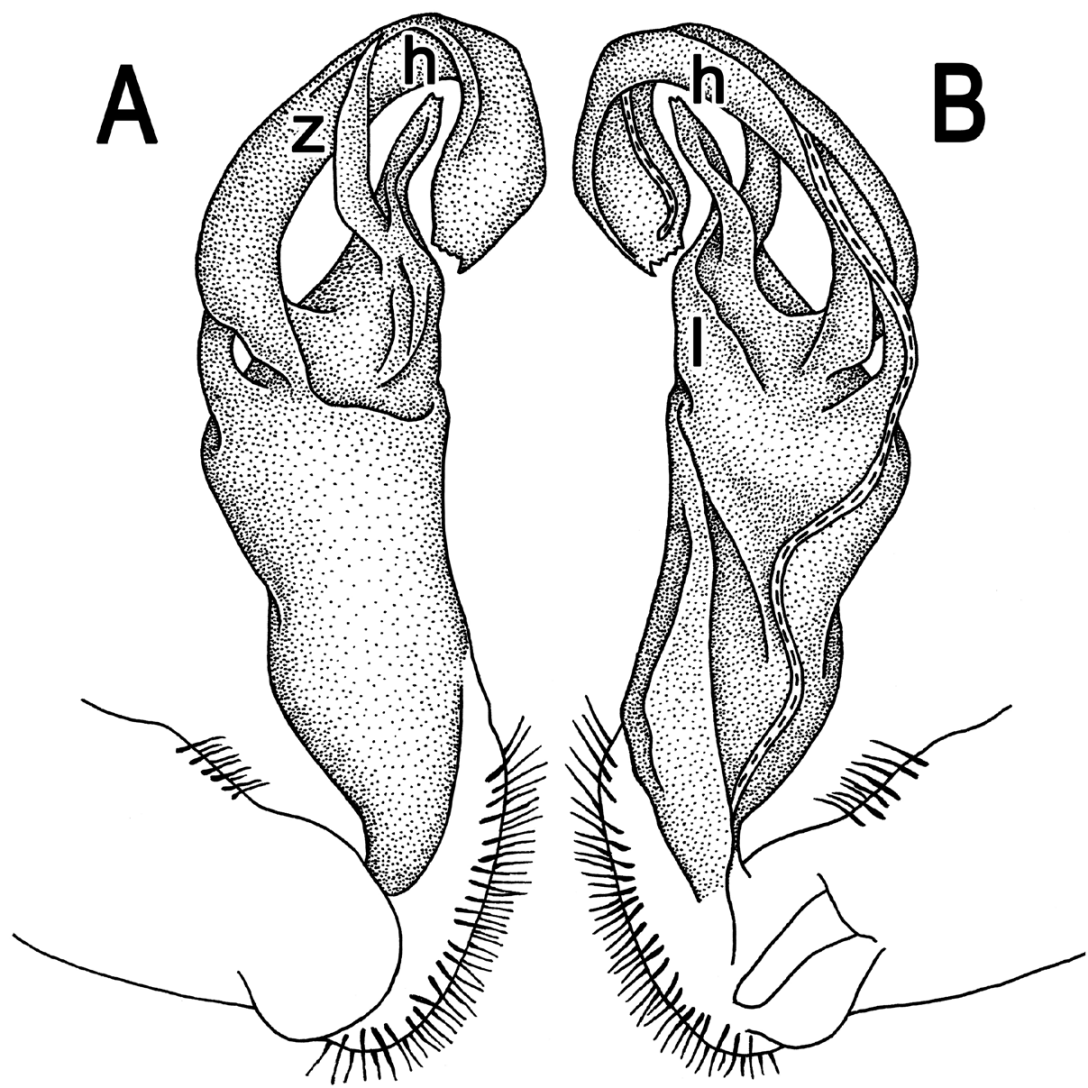
*Tylopus mutilatus* (Attems, 1953), ♂ syntype; **A, B** right gonopod, lateral and mesal views, respectively. Depicted not to scale.

Gonopod (Fig. 14) rather simple. Coxa long and slender, with several setae distodorsally. Prefemur densely setose, nearly 1/3 as long as femorite + “postfemoral” part. Femorite stout, slightly curved, slightly enlarged distad, showing a mesal groove, “postfemoral” part demarcated by an oblique lateral sulcus; lobe **l** simple; process **h** long, rather simple, slightly curved, tip small and bifid; process **z** high, slightly curved, tip acute; solenophore long and slender, typically coiled, tip microdenticulate.

##### Remark.

This species was described both from Luang Prabang, Xieng Kuang, Laos and Pic de Langbiang (Mount Langbian), Lamdong Province, Vietnam (Attems 1953). Golovatch (1984) redescribed and illustrated only a gonopod, but the locality remained unclear. As all our attempts at locating a torso of *Tylopus sigma* in the collection of the Naturhistorisches Museum Wien, Austria had failed, we could only revise the very same right gonopod mounted on a slide. Fortunately, the gonopod is easily distinguished from congeners.

### Key to the species of *Tylopus* currently known to occur in Thailand, chiefly based on ♂ characters:

**Table:** 

1	Most ♂ prefemora clearly swollen laterally (Fig. 5C)	2
–	All ♂ prefemora normal, not bulged laterally (Figs 2E, F, 7C)	9
2	Sternal lamina between ♂ coxae 4 divided into two cones (Fig. 1I, J)	5
–	Sternal lamina between ♂ coxae 4 single, not divided (Figs 4I, J, 6I, J)	3
3	Gonopod processes **z**, **r** and **m** present	*Tylopus coriaceus*
–	Gonopod processes **z**, **r** and **m** absent	4
4	Tarsal bushes on ♂ legs present, from legs pair 3 with tubercles on tarsi, tibiae and femora	*Tylopus degerboelae*
–	Tarsal bushes on ♂ legs absent, starting from legs 9 with tubercles on femora and following podomeres	*Tylopus pallidus*
5	Smaller species: body width less than 2.5 mm. Metaterga with evident oblong ridges on both anterior and posterior halves	*Tylopus corrugatus* sp. n.
–	Larger species: body width more than 2.5 mm. Metaterga with small oblong ridges to faint knobs only on posterior half	6
6	Sternal lamina between ♂ coxae 4 fully divided into paramedian knobs (Fig. 1I, J)	7
–	Sternal lamina between ♂ coxae 4 with a deep median notch (Fig. 4I, J)	8
7	Gonopod process **h** high, stout and strongly helicoid. Pleurosternal carinae missing on segments 18–19	*Tylopus asper*
–	Gonopod process **h** rather small, slender and subdentiform. Pleurosternal carinae missing on segments 15–19	*Tylopus pulvinipes*
8	Coloration with a pattern of a contrasting dark brown inverted triangle at anterior edge of metaterga. Gonopod process **z** absent. Legs of ♂ segment 10 multituberculate ventrally only on femora	*Tylopus trigonum* sp. n.
–	Coloration uniformly pale. Gonopod process **z** present. Legs of ♂ segment 10 multituberculate ventrally on femora, postfemora, tibiae and tarsi	*Tylopus subcoriaceus*
9	Metaterga without evident setiferous tubercles, only sometimes with very small, rudimentary wrinkles or knobs	10
–	Metaterga with evident setiferous tubercles	16
10	Midbody metaterga more than 4.1 mm wide	11
–	Midbody metaterga less than 3.9 mm wide	12
11	Gonopods (Fig. 5A, B) with process **z** prominent and serrate along distal margin, whereas process **h** a strong hook	*Tylopus parahilaroides* sp. n.
–	Gonopod with a short lobe **z**, whereas process **h** very small	*Tylopus grandis*
12	Both processes **h** and **z** of gonopod spiniform	*Tylopus bispinosus*
–	Gonopod different	13
13	Gonopod process **h** subflagelliform, process **m** extremely long and prominent	*Tylopus extremus*
–	Gonopod different	14
14	Gonopod process **h** a strong hook with a distally serrate process **z**; process **m** extremely long and prominent	*Tylopus similirugosus*
–	Gonopod different	15
15	♂ legs shorter, ca 1.2–1.3 times as long as body height. Gonopod lobe **l** velum-shaped and supplied with two denticles; process **z** short and knife-shaped while process **h** rudimentary	*Tylopus veliger*
–	♂ legs longer, ca 1.6–1.7 times as long as midbody height. Gonopod process **z** small, placed closer to base of process **h**	*Tylopus parajeekeli*
16	Most metaterga with a pattern of 2+2 and 2+2 setiferous tubercles in two rows, rear row somewhat less strongly developed than fore one	*Tylopus doriae*
–	Most metaterga with rear row of setiferous tubercles or wrinkles more strongly developed than fore row, the latter (next to) wanting	17
17	Transverse sulcus on metaterga starting from segment 4, either fully or almost fully developed there, always fully developed starting from segment 5	18
–	Transverse sulcus on metaterga starting only from segment 5	20
18	Paraterga 2 rather broadly rounded caudolaterally. Gonopod relatively simple, process **h** poorly developed, no additional outgrowths near base	*Tylopus affinis*
–	Paraterga 2 pointed caudally. Gonopods more complex	19
19	Coloration dark brown, without cingulate pattern. Sternal lamina between ♂ coxae 4 low and distinctly bimodal. Gonopods with tooth **z** prominent and serrate along distal margin	*Tylopus rugosus*
–	Coloration pale, with a cingulate pattern. Sternal lamina between ♂ coxae 4 high, subquadrate. Gonopod tooth **z** smaller and spiniform	*Tylopus semirugosus*
20	Pattern of tergal setation on segments 18 and/or 19: 2+2 and 5+5 in two rows	21
–	Pattern of tergal setation at least on segments 5–19: 2+2 and 4+4 in two rows	24
21	Pattern of tergal setation 2+2 and 5+5 on both segments 18 and 19. Paraterga 2 pointed caudally. Epiproct with pre-apical incisions very close to apical knobs. Sternal lamina between ♂ coxae 4 an unusually low and even ridge. Adenostyles on midbody ♂ postfemora and, to a lesser extent, tibiae exceptionally prominent	*Tylopus poolpermorum*
–	Pattern of tergal setation 2+2 and 5+5 only on segment 19. Paraterga 2 more or less narrowly rounded. Pre-apical incisions on epiproct better removed from tip. Sternal lamina between ♂ coxae 4 concave medially. Ventral adenostyles on ♂ legs less prominent	22
22	Body smaller: width ca 2.0 mm. Sternal lamina between ♂ coxae 4 as a pair of separate, setiferous tubercles. Ventral adenostyles on ♂ legs almost missing. Gonopods without any outgrowth near base of process **h**	*Tylopus haplorugosus*
–	Body larger: width over 3.0 mm. Sternal lamina between ♂ coxae 4 single. Ventral adenostyles on ♂ legs more prominent. Gonopod with a spine near base of process **h**	23
23	Sternal lamina between ♂ coxae 4 high, emarginate. Adenostyles on ♂ postfemora and tibiae well-developed. Gonopods rather simple, process **z** inconspicuous	*Tylopus allorugosus*
–	Sternal lamina between ♂ coxae lower, slightly concave. Adenostyles on ♂ postfemora and tibiae less strongly developed. Gonopods more complex, process **z** long and large	*Tylopus perarmatus*
24	Paraterga 2 pointed caudally. Sternal lamina between ♂ coxae 4 exceptionally densely setose, low, concave ventrally. Gonopods with a medium-sized process **h** and a smaller lobular **z** at base of **h**	*Tylopus jeekeli*
–	Paraterga 2 more or less narrowly rounded caudally. Sternal lamina between ♂ coxae 4 higher and less strongly setose. Gonopod outgrowths **h** and **z** either almost wanting or very large	25
25	Sternal lamina between ♂ coxae 4 with a straight ventral margin. Pleurosternal carinae poorly developed, in ♂ slightly projecting caudad beyond rear margin only until segments 8–10	26
–	Sternal lamina between ♂ coxae 4 slightly concave ventrally. Pleurosternal carinae better developed, in ♂ slightly projecting caudad beyond rear margin at least until segment 15	27
26	Body smaller: width up to 3.1–3.2 mm. Mid-dorsal line very clear on both halves of metaterga. Gonopods relatively simple, with both **h** and **z** almost wanting	*Tylopus hoffmani*
–	Body larger: width 4.0–5.3 mm. Mid-dorsal line not so well-developed at least on rear halves of metaterga. Gonopods more complex, with both **h** and **z** very conspicuous	*Tylopus baenzigeri*
27	Metatergum 19 slightly rugulose posteriorly. Calluses on segment 2 with three, on following paraterga with two, incisions. Gonopods extremely complex, with numerous spiniform outgrowths	*Tylopus perplexus*
–	Metatergum 19 entirely smooth. Calluses with two or three incisions on poreless and poriferous paraterga, respectively. Gonopod less strongly differentiated	*Tylopus amicus*

## Conclusions

**Map 1.**
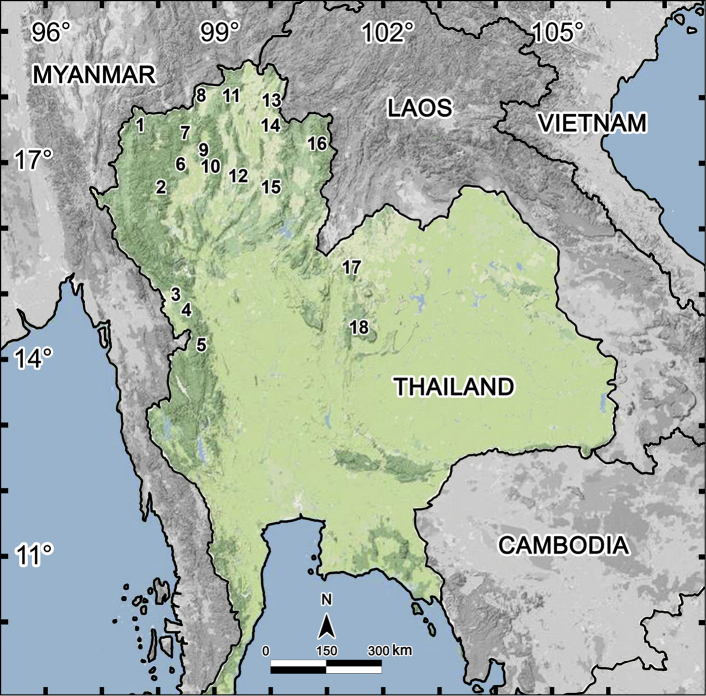
Distribution of *Tylopus* species in Thailand (29 species): **1** Pha Mon Cave: *Tylopus grandis* Likhitrakran et al., 2010 **2** Doi Inthanon: *Tylopus affinis* Golovatch & Enghoff, 1993, *Tylopus allorugosus* Golovatch & Enghoff, 1993, *Tylopus asper* Golovatch & Enghoff, 1993, *Tylopus degerboelae* Golovatch & Enghoff, 1993, *Tylopus haplorugosus* Golovatch & Enghoff, 1993, *Tylopus jeekeli* Golovatch & Enghoff, 1993, *Tylopus perarmatus* Hoffman, 1973, *Tylopus prosperus* Golovatch & Enghoff, 1993, *Tylopus parajeekeli* Likhitrakran et al., 2010, *Tylopus corrugatus* sp. n. **3** Ban Mussoe: *Tylopus semirugosus* Golovatch & Enghoff, 1993 **4** Pa Wai Waterfall: *Tylopus trigonum* sp. n. **5** Umphang District: *Tylopus bispinosus* Likhitrakran et al., 2010 **6** Doi Suthep: *Tylopus affinis* Golovatch & Enghoff, 1993, *Tylopus allorugosus* Golovatch & Enghoff, 1993, *Tylopus baenzigeri* Golovatch & Enghoff, 1993, *Tylopus degerboelae* Golovatch & Enghoff, 1993, *Tylopus doriae* (Pocock, 1895), *Tylopus hoffmani* Golovatch & Enghoff, 1993, *Tylopus jeekeli* Golovatch & Enghoff, 1993, *Tylopus perarmatus* Hoffman, 1973, *Tylopus similirugosus* Golovatch & Enghoff, 1993, *Tylopus subcoriaceus* Golovatch & Enghoff, 1993 **7** Doi Chiang Dao: *Tylopus degerboelae* Golovatch & Enghoff, 1993, *Tylopus perarmatus* Hoffman, 1973, *Tylopus rugosus* Golovatch & Enghoff, 1993 **8** Doi Pha Hom Pok: *Tylopus amicus* Golovatch & Enghoff, 1993, *Tylopus pallidus* Golovatch & Enghoff, 1993, *Tylopus perplexus* Golovatch & Enghoff, 1993, *Tylopus poolpermorum* Golovatch & Enghoff, 1993, *Tylopus extremus* Likhitrakarn et al., 2010 **9** BuathongWaterfall: *Tylopus rugosus* Golovatch & Enghoff, 1993 **10** Doi Phatang: *Tylopus degerboelae* Golovatch & Enghoff, 1993, *Tylopus perarmatus* Hoffman, 1973 **11** Ban Pang Rim Kon: *Tylopus perarmatus* Hoffman, 1973 **12** Thum Pha Thai: *Tylopus perarmatus* Hoffman, 1973 **13** Phucheefah: *Tylopus perarmatus* Hoffman, 1973 **14** Nam Min Waterfall: *Tylopus perarmatus* Hoffman, 1973 **15** Tham Pha Nang Khoi: *Tylopus perarmatus* Hoffman, 1973 **16** Ton Tong Waterfall: *Tylopus veliger* Likhitrakarn et al., 2010 **17** Phuluang Wildlife Sanctuary: *Tylopus parahilaroides* sp. n. **18** Phu Kheio: *Tylopus coriaceus* Golovatch & Enghoff, 1993, *Tylopus pulvinipes* Golovatch & Enghoff, 1993.

Of a total of 55 species of *Tylopus* known now, Thailand supports as many as 29, followed by Vietnam (18 species), southern China (six species), Laos and Myanmar (two species each). The distributions of *Tylopus* spp. in Thailand, most of which are endemic to the country, are shown in Map 1.

Almost all *Tylopus* species appear to be confined to montane forest habitats. In Thailand, *Tylopus* have only been taken from localities exceeding 500 m in elevation, except for Tham Pha Nang Khoi (275 m a.s.l) which solely supports the especially widespread *Tylopus perarmatus*. In contrast, Doi Inthanon and Doi Suthep mountains each harbour as many as 10 species (Table 1), one of the highest values for congeners per local faunule among all Diplopoda, following perhaps the *madeirae*-group of *Cylindroiulus* endemic to Madeira, Portugal (29 spp., Enghoff 1982, Read 1989) or *Dolichoiulus* on Teneriffe, Canary Islands, Spain (21 spp., Enghoff 1992, 2012).

There is no doubt that more species of *Tylopus* will be found in the future, as at least the faunas of southern China, Myanmar, Laos and even Vietnam seem to be quite underrepresented compared to Thailand, while Cambodia is a completely blank area.

## Supplementary Material

XML Treatment for Tylopus corrugatus

XML Treatment for Tylopus parahilaroides

XML Treatment for Tylopus trigonum

XML Treatment for Tylopus nodulipes

XML Treatment for Tylopus hilaris

XML Treatment for Tylopus sigma

XML Treatment for Tylopus mutilatus
